# Is progress in clinical reproductive medicine happening fast enough?

**DOI:** 10.1080/03009734.2020.1734991

**Published:** 2020-05-04

**Authors:** Georg Griesinger

**Affiliations:** Department of Gynecological Endocrinology and Reproductive Medicine, University Hospital of Schleswig-Holstein, Lübeck, Germany

More than 8 million babies have been born from *in vitro* fertilization (IVF) around the world since Louise Brown’s birth in 1978. The number of IVF treatment cycles has globally increased to more than 2 million per year (1). In Europe, treatment numbers lately grew by an estimated 7% per year, with Denmark and Belgium at the top of the league table, each offering more than 2500 IVF treatment cycles per million people (2). Assisted reproduction has arrived to take its place within mainstream medicine. Infertility has also lost its social stigma: a quick google search on the keywords infertility & support and infertility & stigma yields 119 million and 2.3 million results, respectively, a 51:1 ratio in favour of support.

Important milestones in assisted human reproduction were the cryopreservation of embryos (1984) and oocytes (2003), the introduction of ICSI (1992), the use of testicular sperm (1993), preimplantation genetic testing (1989), ovarian tissue transplantation (2000), uterus transplantation (2015) and ‘three-parent babies’ after mitochondrial transfer (2016). These techniques, together with the availability, on a global scale, of gamete donation and gestational surrogacy, provide a wide range of treatment options for people faced with subfertility or infertility. The chance of having a full-term, normal birth weight and singleton live birth per IVF cycle using fresh embryos from non-donor eggs for women younger than 35, according to US figures from 2015, is 21.3% (3). In women 38–40 years of age, this number drops to 11.1% (3). For Australia, a live birth rate of 23.3% for fresh autologous embryo transfers has recently been reported (4). Of note, it is estimated that, for example, in Denmark, only approximately 50% of infertile couples initiating IVF treatment will eventually have a baby within a 5-year span (5).

## IVF: a success story with some obvious limitations

While the successes of modern assisted reproductive technology (ART) are obvious, so are the limitations: a high treatment failure rate per cycle, widespread use of IVF on a purely empirical basis, a lack of management options in case of age-related ovarian depletion, medical risks associated with ovarian stimulation and oocyte retrieval, a high psychological burden associated with infertility itself but also with the treatment, high financial costs especially for couples not on reimbursement schemes, a high multiple pregnancy rate, insufficient knowledge on the long-term risks of some aspects of IVF treatment.

New technologies are therefore constantly proposed to improve IVF outcomes (6). The list of currently offered, presumably beneficial adjuncts is seemingly endless: from pharmacological add-ons (dehydro-epiandrostenedione, growth hormone, testosterone, coenzyme Q10, heparin, low-dose aspirin, vasodilators, myo-inositol, etc.) to laboratory technology (sperm DNA fragmentation testing, sperm selection procedures, time-lapse embryo monitoring, preimplantation genetic screening, assisted hatching, endometrial injury, embryo adherence compounds, etc.) to the different approaches to regenerate, rejuvenate or reactivate germ cells in the human ovary (intra-ovarian injection of calcium gluconate-activated autologous platelet-rich plasma, the use of autologous mitochondrial transfer into oocytes, the generation of artificial gametes from putative ovarian stem cells or the *in vitro* activation of dormant follicles by hippo-signalling disruption, etc.).

Of note, all innovations to IVF treatment have to be judged from one perspective: will a new technology help more couples have a baby with less effort, less burden, less financial costs, and less parental and foetal risk? The recent history of reproductive medicine, however, indicates that very few, if any, new developments have been introduced into clinical practice after thorough and rigorous clinical validation. That is why, more than 20 years after the introduction of some of the technologies named above, there is still no clear picture of the clinical utility.

## RCTs: how much have they really helped?

It is commonly accepted that randomized controlled trials (RCTs) are best suited to evaluate the effectiveness of interventions. When the outcome of interest is binary (in reproductive medicine it is, broadly speaking, ‘baby or no baby’), large sample sizes are necessary to detect clinically relevant effects with sufficient confidence. In a recent systematic review on RCTs and meta-analyses published within the field of reproductive medicine (7), it was found that the vast majority of RCTs are underpowered for this purpose. Even meta-analyses, synthesizing multiple RCTs, could not make up for this. Within the Cochrane library, not a single sufficiently powered RCT could be identified, and only 2% of the meta-analyses were large enough to detect a difference of 5% in live birth rate (e.g. an increase from 25–30%) with a given intervention (7). Moreover, researchers have so far been using differently assessed and defined outcomes, which contributes to the inability to compare and combine individual RCTs (8). Despite the widespread call for RCTs in our field (9), the progress achieved through performing RCTs as we did so far has been, taking a sober view at it, rather limited.

## Why is progress in IVF so slow?

RCTs are a powerful instrument, but collaborative efforts are needed for sufficiently large trials. While both collaboration and competition are essential for scientific progress, the IVF world appears to be occupied with competition on the economic side of ART provision rather than the science of human reproduction. IVF is seeing industrialization of medical services at rapid speed and large scale and conglomeration of individual treatment centres into large networks, often under one brand name and often funded by private equity. The necessity of constant economic growth under such circumstances necessitates competition for patients in need of services to solve their infertility problems or expanding existing treatments to newly identified groups of patients (‘social freezing’ being a good example of the latter). Patients use social media and the internet as a knowledge resource, but the information available freely on the net is often of unclear validity or biased outright by commercial interest. Doctors try to advertise their services on the internet either openly (where legally permissible) or hidden within press releases about putative novel techniques and treatment breakthroughs. All these driving forces tempt researchers and practicing clinicians to jump on the moving bandwagon of the new and superficially promising treatment option often too early. The moment that money is made from a new intervention, the incentive for further research with the risk of disproving initial expectations is often gone.

## What next as a science policy in human reproductive medicine?

Certification of IVF centres may therefore in the future take different levels of scientific engagement within the field as a prerequisite. Borrowing from oncology, a distinction could be made between simple care providers, specialized centres (e.g. PGD-M centres or fertility preservation centres) and comprehensive centres with major research aims. Certification schemes of centres on a national or European level could consider, within the catalogue of requirements, different levels of research activity as a requirement for admittance to general care as well as admittance to more advanced or experimental treatments. For example, in a first step, it could be a requirement for all IVF centres to include a given fraction of the overall treated population in registered trials. Of note, the implementation of quality control systems in our field has not been based on voluntary action alone, given the ramifications and costs for implementing and auditing these systems. Why would that not also work for research?

Reproductive medicine and the research priorities identified within the field need to be put back into the focus of public interest, and public funding is of essence for research networks to be built and large collaborative trials to be conducted. For achieving that, we will not only need to beat the drum for our field, we will also need to demonstrate that we are structurally capable and inherently professionally motivated to run these trials.

Finally, we must look beyond the RCT. As an educated guess, nearly all IVF centres in the industrialized world are using electronic patient files. We have large national and international IVF data registries by now already. In other words, we are already sitting on a mountain of data, but have not been making intelligent use of these data. Observational data can be an easily accessible and cheap method to look at the safety and effectiveness of different treatment strategies. Moreover, large-scale observational data stem from real-world circumstances and therefore include those patients that RCTs typically exclude. Sophisticated statistical methods including multivariable logistic regression analysis and propensity-matched analysis will be needed, but the establishment of collaborations with biostatisticians and epidemiologists may be, in many instances, an easier, faster and much cheaper option as compared to the design, conduct and analysis of large-sized RCTs.

New technologies, new drugs and new treatments will continue to enter our field. These novelties will improve the efficiency, effectiveness, quality, sustainability, safety and/or affordability of reproductive medicine, but only if we as a medical community manage, in a timely manner, to sort out the useless, and then focus our attention and power on the promising.
